# Switching Regulator Based on a Non-Inverting Step-Down/Up DC–DC Converter for Lithium-Ion Battery Applications

**DOI:** 10.3390/mi14061144

**Published:** 2023-05-29

**Authors:** Juan Antonio Villanueva-Loredo, Ma Guadalupe Ortiz-Lopez, Jesus Leyva-Ramos, Luis Humberto Diaz-Saldierna

**Affiliations:** 1 Control and Dynamical Systems Division, The Institute for Scientific and Technological Research of San Luis Potosi (IPICYT), Camino a la Presa de San Jose 2055, San Luis Potosi 78216, Mexico; juan.villanueva@ipicyt.edu.mx (J.A.V.-L.); jleyva@ipicyt.edu.mx (J.L.-R.); ldiaz@ipicyt.edu.mx (L.H.D.-S.); 2 ITMA Department, Universidad Politecnica de San Luis Potosi, Urbano Villalón 500, Col. La Ladrillera, San Luis Potosi 78369, Mexico

**Keywords:** step-down/up converter, switching regulator, loop-shaping control, lithium-ion batteries

## Abstract

A regulator based on a converter with step-down/up characteristics is discussed in this paper, which is suitable for processing energy from a lithium-ion battery pack, where the voltage fluctuates from above or below the nominal value. However, this regulator can also be used for applications such as unregulated line rectifiers and renewable energy sources, among others. The converter consists of a non-cascaded interconnection of boost and buck–boost converters such that part of the input energy is transferred directly to the output without reprocessing. Furthermore, it has a non-pulsating input current and a non-inverting output voltage, making it easier to feed the power to other devices. For control purposes, non-linear and linear converter models are derived. The transfer functions of the linear model are used to implement the regulator using a current-mode control scheme. Finally, experimental results for a nominal output voltage of 48 V at 500 W are obtained for the converter in open-loop and closed-loop tests.

## 1. Introduction

Step-down and step-up converters are widely used topologies in conventional switching converters [1]; however, with the development of new technologies, applications have arisen where DC–DC converters, including control schemes, that provide both characteristics are necessary.

Among these are systems and vehicles powered by fuel cells [2], by batteries that include both electric vehicles and more electric aircraft (MEA) [3,4,5], as well as mobile telecommunications equipment [6,7,8]. All of them require an interface that regulates the output voltage of the power supply, which fluctuates around its nominal value and supplies a load.

**Figure 1:**
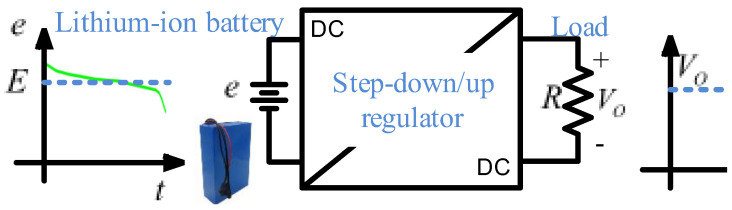
Regulation of the output voltage of LIB pack to a load.

Lithium-ion batteries (LIBs) are used as power sources in electric vehicles, digital cameras, and many other portable devices such as mobile phones, laptops, and medical equipment. However, one of the most common problems is that LIBs require a connection to a voltage regulator to maintain a constant output voltage [9], as shown in Figure 1. A typical case is IoT (Internet of things) devices that need a bus of 3.3 V to regulate the LIB fluctuation from 1.8 V to 5 V [10], or in portable bioelectric equipment such as the cardiac pacemaker and capsule endoscopy [11] where 3.6 V are regulated from a battery fluctuation of 2.5 V to 5 V. Another is the regulator based on a SEPIC converter with a switched inductor proposed in [12] to maintain a fluctuating voltage of 18–24 V to a constant value of 21 V for a laptop.

Lithium batteries are preferred over other rechargeable batteries with similar power characteristics because they have a higher energy density. In addition, lithium is a highly reactive element, and LIBs are lighter than other batteries [13]. Connecting LIBs in a pack with different serial or parallel arrangements gives different nominal voltages and current capacities [14]. Power source requirements in many areas have increased over time in electronic systems that have caused the battery requirement of 12 V or 24 V to change to 48 V, such as in powering data centers [15], telecommunications systems, electric vehicles [16], as well as storing energy from renewable sources [17,18]. The nominal 48 V of an LIB pack varies from 40 V when the battery is discharged to 56 V when fully charged. Then, to maintain the constant nominal output voltage, the battery requires regulators based on DC–DC converters.

An essential issue in an LIB pack is the behavior of its output current. High ripple currents, faster charge and discharge periods, or high harmonic currents might reduce the lifetime of the LIB pack and increase heating and the overpotential built-up, negatively affecting the efficiency [19,20,21]. Therefore, the converter selected to implement a regulator must exhibit step-down/up characteristics and a non-pulsating input current. The latter considerably reduce the current ripple demanded from the battery, guaranteeing the best performance of the LIB.

The following review of different topologies of step-down/up converters presented in the technical literature that could suit this application is given. The simplest is the conventional buck–boost converter; however, it has an inverting output voltage, making it challenging to connect other devices to the same power source [22]. Therefore, many non-inverting buck–boost converter topologies have been proposed. Based on structures with zero-voltage switching, in [23] is presented a converter with continuous input current but manages a low power of 35 W; meanwhile, in [24], a converter with coupled inductors and a soft switching scheme is proposed, with the drawback of non-continuous input current. Moreover, this class of topologies using synchronous switching is presented in [25,26]. In the first one, the converter has an efficiency near 93% but with a low power of 60 W and a non-continuous input current. The second converter is used only as a buck or boost converter, aiming to increase efficiency. Finally, an interleaving non-inverter buck converter structure for low-power applications is proposed in [27] with the drawback of a non-continuous input current, making it unsuitable for the intended application. Meanwhile, in [28], another interleaving non-inverter structure with three output stages to improve the voltage gain is analyzed. Although this converter has a non-pulsing input current, it also has a very high output gain that is inadequate for regulating an output voltage around an operating value. Other alternatives are the Cuk or SEPIC converters; they have a non-pulsating input current and step-down/up the input voltage capabilities. However, they require semiconductors with a high current stress capacity and high capacitance in the transfer stage [29]. Quadratic step-up/down converters handle a higher voltage gain as $D^{2}/{\left(1-D\right)}^{2}$ [30] or $D/{\left(1-D\right)}^{2}$ [31]; however, a simple voltage gain of D/(1−D) is enough to regulate the voltage fluctuations of an LIB to its nominal value, and both converters are used only to reduce or increase an output voltage. Finally, isolated converters might be considered, but the isolation transformer makes them less efficient, more expensive, and bulkier than the last options. Another alternative solution could be the cascaded connection of boost and buck converters; however, the power processed by the boost converter will be processed by the buck converter resulting in poor efficiency.

**Figure 2:**
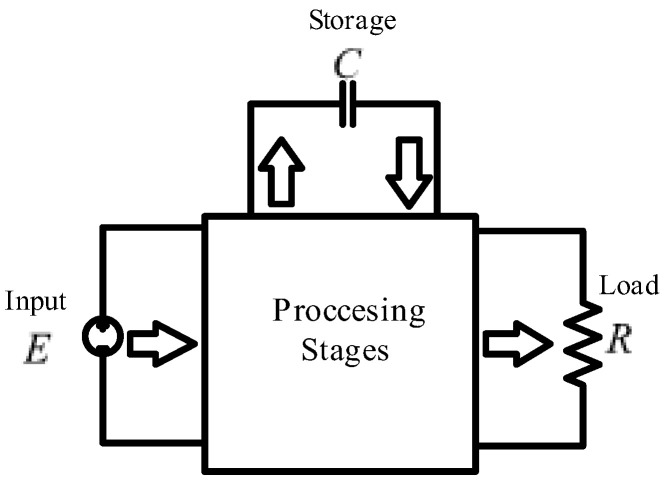
Scheme of the principle of operation of reduced redundant power processing.

Based on the above, it is concluded that a non-cascading connection of simple converters could be a better solution. In these topologies, part of the energy is processed only by one converter; thus, they have reduced redundant power processing (r^2^p^2^) [32,33]. To carry out the analysis, a converter made up of two stages can be represented by three ports, as shown in Figure 2: the input port or source that supplies energy, the storage port or capacitor that stores energy but also delivers it, and the output port or load that only absorbs power.

In a cascade connection of converters, the first stage processes all the input energy and then delivers it to the storage port; and the second stage processes the energy from the storage port and sends it to the output. Therefore, the two stages process all the energy. However, when part of the power taken from the source goes directly to the output port, that is, it is only processed by one stage, there is a converter with a non-cascade structure or reduced redundant power processing converter.

**Figure 3:**
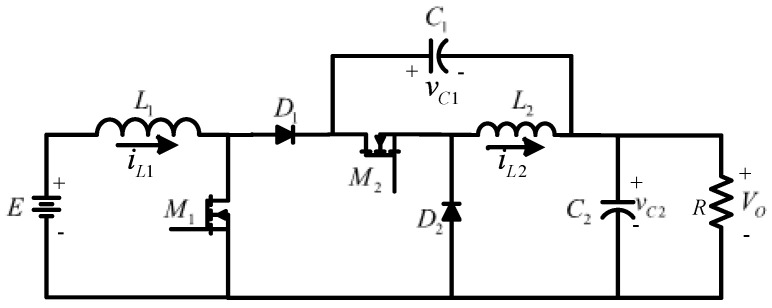
Resulting topology of non-inverting step-down/up converter.

There are fifteen configurations in how to interconnect two converters with r^2^p^2^. A deep study about which configurations are feasible and non-feasible is developed using the buck, boost, and buck–boost converters [34,35]. Following the possible configurations, a converter based on a non-cascading interconnection with two stages is built; the first stage is a boost converter, and the second is a buck–boost converter, as shown in Figure 2. The structure can be simplified for better visualization, as shown in Figure 3, in which E represents the input voltage and *V_O_* the output voltage. The transfer capacitor is represented by $C_{1}$, and the output capacitor by $C_{2}$. The inductor of the first stage is $\;L_{1}$, and the inductor of the second stage is $L_{2}$. The MOSFETs $M_{1}$ and $M_{2}$ are the active switches operating simultaneously; $D_{1}$ and $D_{2}$ are the diodes; and R is the load.

Thus, the resulting converter to be used in the regulator has the following advantages:

- Step-down/up voltage with the non-cascade interconnection of stages, that increase power processing efficiency;
- Low number of active and passive switches as well as electrical components;
- Non-pulsating input current;
- Non-inverting output voltage;
- The input and output share a common ground.

This paper aims to develop a procedure to design a switching regulator based on a non-inverting step-down/up converter for an LIB pack, as shown in Figure 1. Waveforms of the main variables of the converter, a detailed procedure to obtain its output-to-input-voltage relationship and mathematical expressions for an appropriate parameter selection based on steady-state analysis, and dynamical models to design a control scheme are derived for the converter. Finally, the switching regulator is designed using loop-shaping techniques. The outline of this paper is as follows: In Section 2, the structure of the converter and its operation are studied; then, steady-state and semiconductor stress expressions are obtained, as well as relationships for an adequate selection of components. In Section 3, nonlinear and linear models are developed to describe the dynamical behavior of the converter where the transfer functions of interest are obtained. Section 4 gives expressions to calculate the power losses in each component. In Section 5, a current-mode scheme is proposed to control the converter. In Section 6, open-loop and closed-loop experimental results are shown. Finally, in Section 7, conclusions and remarks are provided.

## 2. Analysis and Design of the Step-Down/Up Converter

### 2.1. Steady State Response

**Figure 4:**
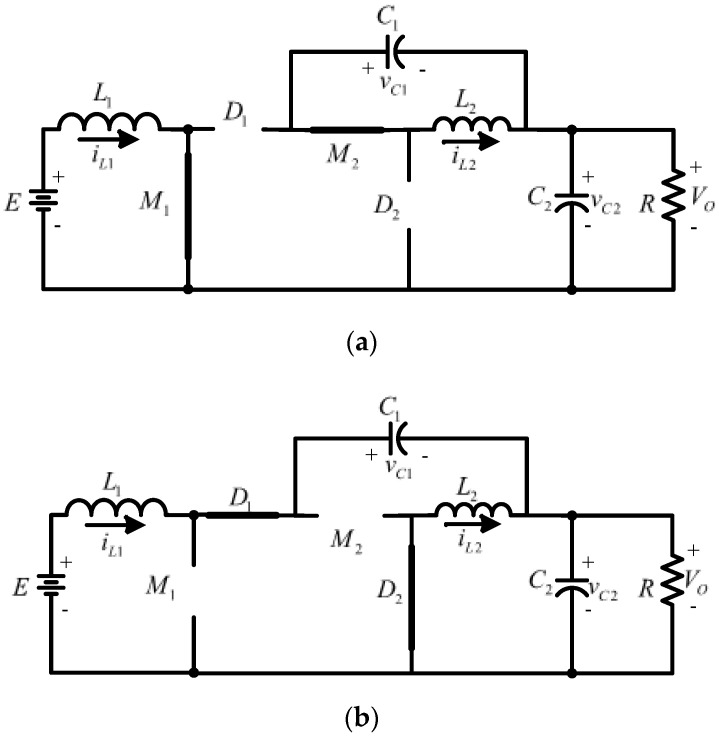
Circuit diagrams of the converter when the active switches M_1_ and M_2_ are: (**a**) ON, and (**b**) OFF.

**Figure 5:**
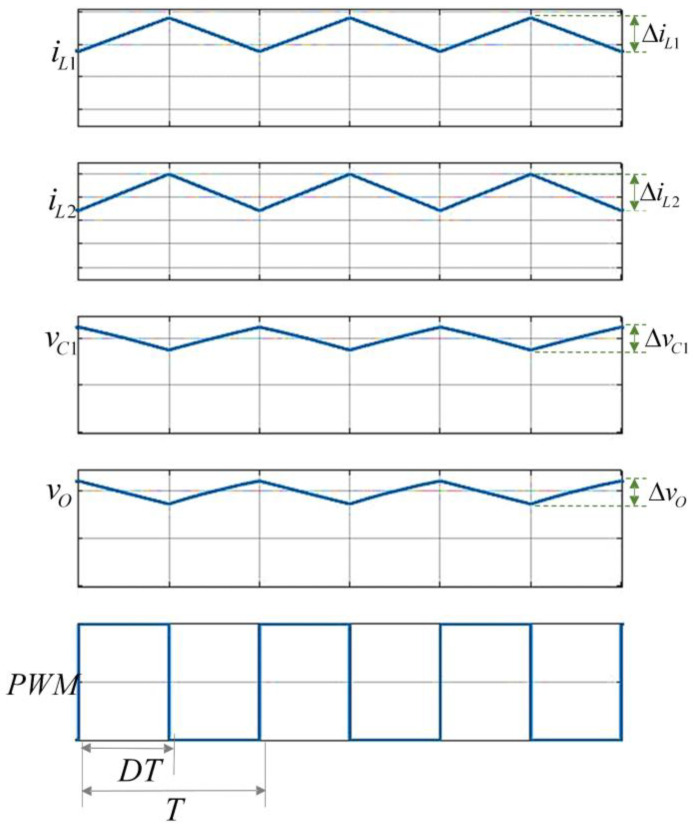
Wave forms of converter variables.

The non-inverting step-down/up converter shown in Figure 3 is assumed to operate in continuous conduction mode (CCM), which means that the inductor currents never decay to zero. The active switches $M_{1}$ and $M_{2}$ operate simultaneously. The electrical circuits are shown in Figure 4 and the resulting wave forms in Figure 5, when they are ON and OFF.

The ON state is shown in Figure 4a, for a time corresponding to DT in which D represents the duty cycle and T the period of a switching cycle of the converter, respectively; $M_{1}\;$ and $M_{2}$ are closed; and $D_{1}$ and $D_{2}$ are reverse biased. It is possible to identify three different parallel connections—the source with inductor $L_{1}$, capacitor $C_{1}$ with inductor $L_{2}$, and capacitor $C_{2}$ with load R—so both inductors charge energy, and both capacitors release energy. Thus, in this condition:

(1) $$V_{L1}=E$$

(2) $$V_{L2}=V_{C1}$$

and the relationship for the voltage of the first inductor can be written as:

(3) $$L_{1}\frac{\Delta i_{L1C}}{DT}=E.$$

Meanwhile, the relationship for the second inductor is given by:

(4) $$L_{2}\frac{\Delta i_{L2C}}{DT}=V_{C1}$$

Therefore, the current ripple of the first inductor during charging is:

(5) $$\Delta i_{L1C}=\frac{EDT}{L_{1}}$$

and for the second inductor is:

(6) $$\Delta i_{L2C}=\frac{V_{C1}DT}{L_{2}}$$

In the OFF state, for a time corresponding to (1−D)T, $M_{1}$ and $M_{2}$ are open, and $D_{1}$ and $D_{2}$ are forward biased; therefore, both inductors discharge and capacitors charge. In this condition:

(7) $$E=V_{L1}+V_{C1}+V_{C2}$$

and

(8) $$V_{L2}=-V_{C2}$$

The relationship for the first inductor voltage is now:

(9) $$L_{1}\frac{\Delta i_{L1D}}{(1-D)T}=E-V_{C1}-V_{C2}$$

and for the second one is:

(10) $$L_{2}\frac{\Delta i_{L2D}}{(1-D)T}=-V_{C2}$$

Therefore, the current ripple of the first inductor during discharging is given as:

(11) $$\Delta i_{L1D}=\frac{(E-V_{C1}-V_{C2})(1-D)T}{L_{1}}$$

and the ripple for second inductor is:

(12) $$\Delta i_{L2D}=\frac{-V_{C2}(1-D)T}{L_{2}}$$

Because in the inductor the charge ripple is equal to the discharge ripple, $\Delta i_{LC}+\Delta i_{LD}=0$ results in the following expression:

(13) $$\frac{EDT}{L_{1}}+\frac{(E-V_{C1}-V_{C2})(1-D)T}{L_{1}}=0$$

which can by simplified as:

(14) $$V_{C1}+V_{C2}=\frac{E}{1-D}$$

By using the same procedure for the second inductor, the following expression is obtained:

(15) $$\frac{V_{C1}DT}{L_{2}}+\frac{-V_{C2}(1-D)T}{L_{2}}=0,$$

which can by simplified as:

(16) $$V_{C1}=\frac{V_{C2}(1-D)}{D}$$

Finally, as $V_{C2}=V_{O}$, the voltage gain results in:

(17) $$\frac{V_{O}}{E}=\frac{D}{1-D}.$$

**Figure 6:**
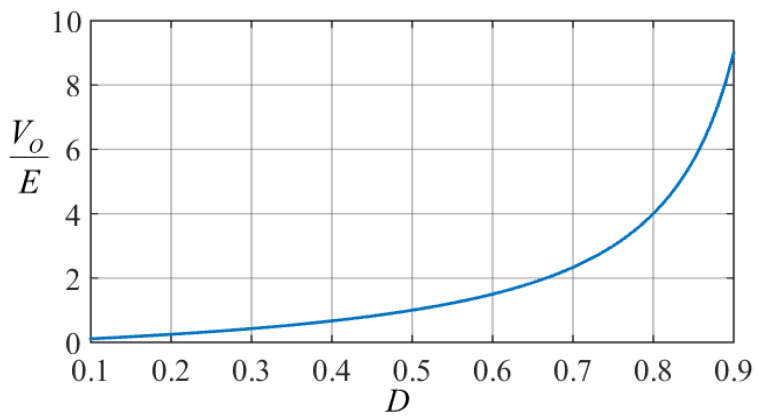
Plot of the voltage gain of the converter.

This expression is plotted and shown in Figure 6. Here, it can be observed that when 0 < *D* < 0.5, the converter demonstrates step-down behavior because $V_{O}/E<1$, and when 0.5 < *D* < 1, the converter demonstrates step-up behavior because $V_{O}/E>1$.

The expressions for the average voltages in capacitors and average currents in inductors can be computed by:

(18) $$V_{C1}=E,$$

(19) $$V_{O}=\frac{DE}{1-D},$$

(20) $$I_{L1}=\frac{D^{2}E}{{\left(1-D\right)}^{2}R},$$

and

(21) $$I_{L2}=\frac{DE}{(1-D)R},$$

The values of inductors and capacitors are computed using the following expressions:

(22) $$L_{1}=\frac{ED}{\Delta I_{L1}f_{S}},$$

(23) $$L_{2}=\frac{ED}{\Delta I_{L2}f_{S}},$$

(24) $$C_{1}=\frac{D^{2}E}{(1-D)\Delta V_{C1}f_{S}R},$$

and

(25) $$C_{2}=\frac{D^{2}E}{(1-D)\Delta V_{O}f_{S}R},$$

where $f_{S}$ is the switching frequency, $\Delta I_{L1}$ and $\Delta I_{L2}$ stand for the inductor current ripples, and $\Delta V_{C1}$ and $\Delta V_{C2}$ stand for the capacitor voltage ripples. For simplicity, the voltage drops caused by the switching devices were neglected. To guarantee the operation in CCM, the inductors must satisfy the following inequalities:

(26) $$L_{1}>\frac{{\left(1-D\right)}^{2}R}{2f_{S}D},$$

and

(27) $$L_{2}>\frac{(1-D)R}{2f_{S}}.$$

If some of the inductors do not satisfy this inequality or the resistance *R* changes considerably, the operation mode of the converter can change to discontinuous conduction mode.

Calculating the voltage and current stress of the semiconductor components is essential. For example, the voltage stress in the active and passive switches are computed by:

(28) $$V_{M1}=V_{M2}=V_{D1_{\;}}=V_{D2}=\frac{E}{1-D},$$

Meanwhile, the current stresses are given by:

(29) $$I_{M1}=\frac{D^{3}E}{{\left(1-D\right)}^{2}R},$$

(30) $$I_{M2}=\frac{D^{2}E}{(1-D)R},$$

(31) $$I_{D1}=\frac{D^{2}E}{(1-D)R},$$

and

(32) $$I_{D2}=\frac{DE}{R}$$

### 2.2. Component Selection

When designing a converter, specifications must be met regarding the values of the voltage ripple in the capacitors and the output voltage, as well as the current ripple in the inductors; these specifications are expressed as percentages. The voltage ripple percentage for the capacitor voltage is given by the relationship $\varepsilon \%=(\Delta V_{Ci}/2V_{Ci})\cdot 100$ with a typical value in a conventional converter [22] between 1% and 2%, and the inductor ripple percentage for the current that flows through them by $\varepsilon \%=(\Delta I_{Li}/2I_{Li})\cdot 100$ with a typical value between 15% and 30%.

By using the expressions given in (18) to (21) and (22) to (25), it is possible to calculate the corresponding values for the inductors and capacitors. The resulting expressions are shown in Table 1.

As stated in Section 2.1, inductors $L_{1}$ and $L_{2}$ must satisfy inequalities (26) and (27) to guarantee CCM.

## 3. Modeling and Dynamical Analysis of Step-Down/Up Converter

The dynamical behaviour of the converter is described through mathematical models. It allows for designing a control strategy so the converter can regulate fluctuations in the input voltage and output load and compensate for the parasitic uncertainties.

First, a bilinear piecewise model is obtained. It uses the electric circuits formed with the ON and OFF states of the active switches shown in Figure 4. Then, equations that describe these circuits are calculated and put together through a binary switching function denoted by *q*, where *q* = 1 represents the ON state while *q =* 0 represents the OFF state. The capacitor voltages and the inductor currents are the four state variables. The resulting model is given by:

(33) $$\left[\begin{array}{c} {\dot{i}}_{L1}\\ {\dot{i}}_{L2}\\ {\dot{v}}_{C1}\\ {\dot{v}}_{O} \end{array}\right]=\left[\begin{array}{cccc} 0 & 0 & -\frac{1-q}{L_{1}} & -\frac{1-q}{L_{1}}\\ 0 & 0 & \frac{q}{L_{2}} & -\frac{1-q}{L_{2}}\\ \frac{1-q}{C_{1}} & -\frac{q}{C_{1}} & 0 & 0\\ \frac{1-q}{C_{2}} & \frac{1-q}{C_{2}} & 0 & -\frac{1}{C_{2}R} \end{array}\right]\left[\begin{array}{c} i_{L1}\\ i_{L2}\\ v_{C1}\\ v_{O} \end{array}\right]+\left[\begin{array}{c} \frac{1}{L_{1}}\\ 0\\ 0\\ 0 \end{array}\right]e$$

Now, the nonlinear average model can be derived [36] using the average value of each state variable represented by a superscript “−” and the average value of the variable *q* represented by the duty cycle *d*. Here, the state variables are multiplied by the duty cycle; therefore, the resulting system is nonlinear:

(34) $$\left[\begin{array}{c} {\dot{\bar{i}}}_{L1}\\ {\dot{\bar{i}}}_{L2}\\ {\dot{\bar{v}}}_{C1}\\ {\dot{\bar{v}}}_{O} \end{array}\right]=\left[\begin{array}{cccc} 0 & 0 & -\frac{1-d}{L_{1}} & -\frac{1-d}{L_{1}}\\ 0 & 0 & \frac{d}{L_{2}} & -\frac{1-d}{L_{2}}\\ \frac{1-d}{C_{1}} & -\frac{d}{C_{1}} & 0 & 0\\ \frac{1-d}{C_{2}} & \frac{1-d}{C_{2}} & 0 & -\frac{1}{C_{2}R} \end{array}\right]\left[\begin{array}{c} {\bar{i}}_{L1}\\ {\bar{i}}_{L2}\\ {\bar{v}}_{C1}\\ {\bar{v}}_{O} \end{array}\right]+\left[\begin{array}{c} \frac{1}{L_{1}}\\ 0\\ 0\\ 0 \end{array}\right]\bar{e}$$

Finally, linearization techniques are applied to describe the dynamics of the converter. Model (34) is linearized around the operating point corresponding to the steady-state values given by (18) to (21). The control signal and four state variables are decomposed into two parts: the nominal average values, which are denoted by upper-case letters, and their corresponding deviations, which are denoted by letters with a superscript “~”. The resulting linearized model is:

(35) $$\left[\begin{array}{c} {\dot{\tilde{i}}}_{L1}\\ {\dot{\tilde{i}}}_{L2}\\ {\dot{\tilde{v}}}_{C1}\\ {\dot{\tilde{v}}}_{O} \end{array}\right]=\left[\begin{array}{cccc} 0 & 0 & -\frac{1-D}{L_{1}} & -\frac{1-D}{L_{1}}\\ 0 & 0 & \frac{D}{L_{2}} & -\frac{1-D}{L_{2}}\\ \frac{1-D}{C_{1}} & -\frac{D}{C_{1}} & 0 & 0\\ \frac{1-D}{C_{2}} & \frac{1-D}{C_{2}} & 0 & -\frac{1}{C_{2}R} \end{array}\right]\left[\begin{array}{c} {\tilde{i}}_{L1}\\ {\tilde{i}}_{L2}\\ {\tilde{v}}_{C1}\\ {\tilde{v}}_{O} \end{array}\right]+\left[\begin{array}{c} \frac{E}{(1-D)L_{1}}\\ \frac{E}{(1-D)L_{2}}\\ -\frac{ED}{{\left(1-D\right)}^{2}RC_{1}}\\ -\frac{ED}{{\left(1-D\right)}^{2}RC_{2}} \end{array}\right]\tilde{d}$$

Using the Laplace transforms in the average linear model (35), the transfer function of the input current ${\tilde{i}}_{L1}$ and the output voltage ${\tilde{v}}_{O}$ with respect to the duty cycle $\tilde{d}$ are computed for control purposes:

(36) $$\frac{{\tilde{i}}_{L1}(s)}{\tilde{d}(s)}=\frac{b_{3}s^{3}+b_{2}s^{2}+b_{1}s+b_{0}}{s^{4}+a_{3}s^{3}+a_{2}s^{2}+a_{1}s+a_{0}},$$

and

(37) $$\frac{{\tilde{v}}_{O}(s)}{\tilde{d}(s)}=\frac{c_{3}s^{3}+c_{2}s^{2}+c_{1}s+c_{0}}{s^{4}+a_{3}s^{3}+a_{2}s^{2}+a_{1}s+a_{0}},$$

where: $a_{3}=\frac{1}{C_{2}R}$, $a_{2}=\frac{{\left(1-D\right)}^{2}(C_{1}L_{1}+C_{1}L_{2}+C_{2}L_{2})+C_{2}D^{2}L_{1}}{C_{1}C_{2}L_{1}L_{2}}$, $a_{1}=\frac{L_{2}{\left(1-D\right)}^{2}+D^{2}L_{1}}{C_{1}C_{2}L_{1}L_{2}R}$, $a_{0}=\frac{{\left(1-D\right)}^{2}}{C_{1}C_{2}L_{1}L_{2}}$; $b_{3}=\frac{E}{L_{1}(1-D)}$, $b_{2}=\frac{E(C_{1}+C_{1}D+C_{2}D)}{C_{1}C_{2}L_{1}R(1-D)}$, $b_{1}=\frac{ED(L_{2}+C_{2}R^{2})}{C_{1}C_{2}L_{1}L_{2}R^{2}(1-D)}$, $b_{0}=\frac{2ED}{C_{1}C_{2}L_{1}L_{2}R(1-D)}$; $c_{3}=\frac{-ED}{C_{2}R{\left(1-D\right)}^{2}}$; $c_{2}=\frac{E(L_{1}+L_{2})}{C_{2}L_{1}L_{2}}$; $c_{1}=\frac{-ED^{2}}{C_{1}C_{2}L_{2}R{\left(1-D\right)}^{2}}$; $c_{0}=\frac{E}{C_{1}C_{2}L_{1}L_{2}}$.

When the numerator and denominator polynomials of both transfer functions were analyzed, it was found that all the poles are located in the left-hand side (LHS) of the s-plane. Furthermore, the transfer function ${\tilde{i}}_{L1}\left(s\right)/\tilde{d}\left(s\right)$ has a minimum phase behavior because it has its zeros in the LHS of the s-plane, and ${\tilde{v}}_{O}(s)/\tilde{d}(s)$ has a non-minimum phase behavior because it has zeros in the right-hand side (RHS) of the s-plane. The non-minimum phase behavior makes this converter more challenging to control because a high-gain controller might cause instability [37].

## 4. Efficiency Analysis

**Figure 7:**
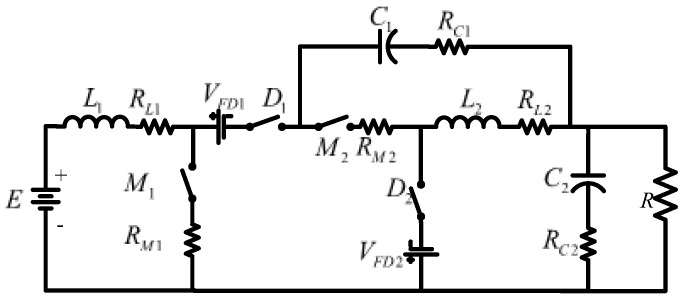
Step-down/up converter with parasitic elements.

One of the most important features of power converters is their efficiency. High efficiency leads to a more reliable converter with a reduced cost and size. The power losses of each converter component are caused by their parasitic elements. The main parasitic element of the step-down/up converter is shown in Figure 7. The corresponding equivalent series resistance of $L_{1}$, $L_{2}$, $C_{1}$, $C_{2}$, $M_{1}$, and $M_{2}$ are represented by $R_{L1}$, $R_{L2}$, $R_{C1}$, $R_{C2}$, $R_{M1}$, and $R_{M2}$, respectively. The voltage drops caused by diodes $D_{1}$ and $D_{2}$ are $V_{FD1}$ and $V_{FD2}$.

Based on the expressions given in [38], the power losses of the step-down/up converter are derived in Table 2. The effective currents $I_{C1\;RMS}$ and $I_{C2\;RMS}$ are used to compute the power losses of $C_{1}$ and $C_{2}$; their values are given by:

(38) $$I_{C1\;RMS}=I_{C2\;RMS}=\frac{2D^{2}E}{(1-D)R}$$

The values of $t_{rr1}$ and $t_{rr2}$ are the turn ON times of $M_{1}$ and $M_{2}$, respectively. The values of $t_{ff1}$ and $t_{ff2}$ are the turn OFF times.

The sum of the individual power losses is the total power loss $P_{L\_T}$ given by:

(39) $$P_{L\_T}=P_{L\_L1}+P_{L\_L2}+P_{L\_C1}+P_{L\_C2}+P_{L\_D1}+P_{L\_D2}+P_{L\_M1}+P_{L\_M2}.$$

If the output power P and the total power losses $P_{L\_T}$ are defined, the estimated efficiency $\eta_{cal}$ can be computed by:

(40) $$\eta_{cal}=\frac{P}{P+P_{L\_T}}$$

The purpose of estimating the efficiency is to obtain a good approximation of the true efficiency of a regulator.

In addition to the losses due to parasitic resistances, in the case of inductors, the losses in the magnetic core must be considered. Therefore, the Ridley–Nace equation given in [39] is used to calculate the magnetic flux in each inductor employing the relationship $B=(L\Delta I_{L})/(zS_{fe})$, in which z is the number of turns and *S_fe_* is the cross-section area of the core [40].

A comparison of the proposed converter with converters for similar applications given in the references of the introduction is provided in Table 3. In addition, the efficiency of the proposed converter is obtained experimentally and shown for different loads in Section 6. The proposed converter has better efficiency than the converters [9,12]. Conversely, the converter [10] has better efficiency but was computed using a software simulator. Moreover, it has a higher total device count, a limited voltage gain range, and a more complex control scheme because it uses six active switches. The efficiency of the converter described in [24] was also obtained using only simulation. Regarding references [24] and [25], these converters have a pulsating current and are unsuitable for the proposed application. Still, they also have a lower efficiency even though, in [25], two efficiencies are obtained, the first for a circuit based on conventional devices and the second using GaN devices, which are used to improve it. Furthermore, quadratic buck–boost converters [30] and [31] are unsuitable for the intended application, and, given their voltage gain, they are used as buck or boost converters exclusively. Finally, it is important to notice that the power managed for the proposed converter 500 W is higher than that handled by all the previously mentioned converters.

## 5. Control Design

The dynamic behavior of switching converters is non-linear; however, linear models can be used for the controller design. Furthermore, the implementation of linear controllers is more straightforward and less expensive than non-linear controllers [41]. Therefore, a linear control scheme is selected.

**Figure 8:**
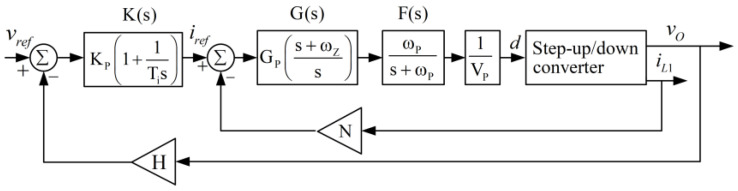
Block diagram of the current-mode controller.

As described in the previous section, ${\tilde{v}}_{O}(s)/\tilde{d}(s)$ has a non-minimum phase behavior but ${\tilde{i}}_{L1}\left(s\right)/\tilde{d}\left(s\right)$ has a minimum phase behavior. Therefore, a current-mode controller is appropriate for this converter. This control scheme is shown in Figure 8, where the input current is feedback in the inner loop and the output voltage in the outer loop [42]. The controller design procedure is based on loop-shaping techniques applied to outer loop gain, which is obtained by the product of its transfer function.

The following conditions should be satisfied for robust stability: 1. The slope at or near crossover frequency must be not more than −20 dB/dec; 2. The gain at low frequencies should be high to enhance steady-state accuracy; and 3. Appropriate gain and phase margins are required. 

The procedure to design each loop, select the gain values [42], and therefore allow to choose the components of the controller circuit, are described below:

### 5.1. Inner Loop

This loop produces a faster transient response and uses a high-gain compensator *G(s)*, a low-pass filter *F(s)*, a sensor gain *N*, and an oscillator ramp $V_{p}$. Here, the average input inductor current follows the reference current.

The expression for this loop is given by:

(41) $$\tilde{d}=\frac{1}{V_{P}}\underset{G(s)}{\underbrace{\left(G_{P}\left(1+\frac{\omega_{Z}}{s}\right)\right)}}\underset{F(s)}{\underbrace{\left(\frac{1}{1+(s/\omega_{P})}\right)}}({\tilde{i}}_{ref}-N{\tilde{i}}_{L1}).$$

For good results, the zero of compensator G(s) has to be placed at least a decade below half of switching frequency *f_S_*, whereas the low-pass filter pole should to be placed either at half of *f_S_* or above. The relationships with controller circuit elements, shown in Section 6, are given by $\omega_{Z}=1/R_{FZ}C_{FZ}$ and $\omega_{P}=(C_{FZ}+C_{FP})/R_{FZ}C_{FZ}C_{FP}$.

The compensator gain is designed so that the inner (current) loop gain has a value close to 10 at frequencies around the zero of *G*(s). The above criterion is satisfied with the following compensator gain:

(42) $$G_{P}<\frac{5V_{P}R{\left(1-D\right)}^{3}}{EDN}.$$

As noticed, the gain *G_p_* has to be robust to changes in the output load. This condition is reached if the *G_p_* value is multiplied by a factor of 8 to 10. In the physical circuit, *G_p_* is adjusted through the relationship of resistances *R_FZ_/R_1F_.*

### 5.2. Outer Loop

This loop is used for output voltage regulation and is designed once the inner loop has been tuned. It uses a PI-controller *K(s)* and a sensor voltage gain *H*, so the output voltage is followed by the reference voltage $v_{ref}$. The expression of this loop is given by:

(43) $${\tilde{i}}_{ref}=K_{P}\underset{K(s)}{\underbrace{\left(1+1/\left(T_{i}s\right)\right)}}({\tilde{v}}_{ref}-H{\tilde{v}}_{O}).$$

The purpose of K(s) is to provide a high gain to the controller at low frequencies; thus, integrative time $T_{i}$ is selected to place the pole of K(s) a decade below *f_s_*, while the gain is selected to obtain the appropriate gain and phase margins of the voltage loop. The expressions for the controller shown in Section 6 are $T_{i}=R_{FC}C_{FC}$, and the gain $K_{p}$ is given by:

(44) $$K_{P}<\frac{2ND}{HR(1-D)}$$

This gain is adjusted in the controller circuit by *R_FC_/R_1C_.* It is important to notice that expressions (42) and (44) provide a first approximation of inner and outer loop controller gains; subsequently, an iterative tuning process has to be carried out to guarantee the appropriate robust stability of the regulator.

## 6. Experimental Results

**Figure 9:**
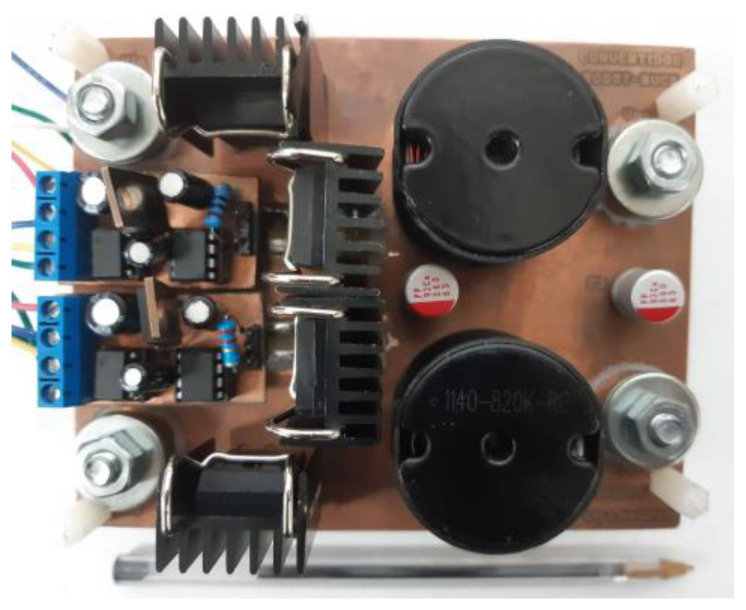
Prototype of the step-down/up converter built in the laboratory.

As mentioned in the introduction, new applications have increased power requirements. The battery requirement of 12 V or 24 V is changing to 48 V. Thus, a prototype designed in the laboratory to validate the theoretical analysis of the step-down/up converter regulates a nominal 48 V output voltage at 500 W, which could come from an LIB pack. The photo of the experimental prototype is shown in Figure 9. Next, the converter parameters are shown in Table 4. Then, using the expressions derived in Section 2, the component values of the converter parameters are chosen and listed in Table 5.

The selected values were used to compute the transfer functions (20) and (21). The resulting poles and zeros of both transfer functions are shown in Table 6. The transfer function input current-to-duty cycle has a minimum phase behavior because all zeros are in the LHS. On the other hand, the transfer function output voltage-to-duty cycle has a non-minimum phase behavior because it has zeros in the RHS.

**Figure 10:**
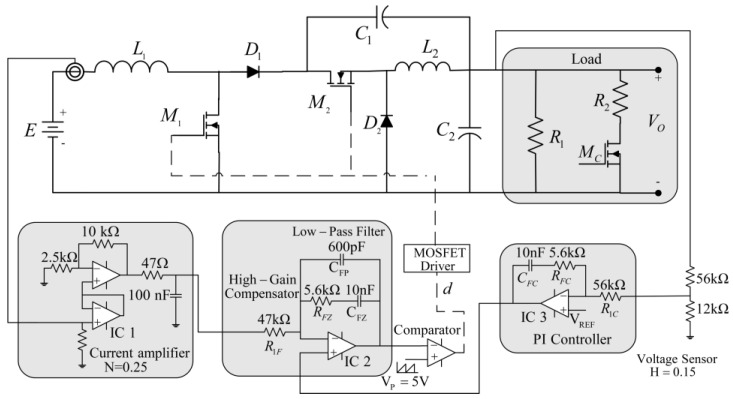
Circuit diagram of the current-mode controller for the switching regulator.

A current-mode controller is designed for the step-down/up converter, which is shown in Figure 10. In the inner current loop, the sensor gain is *N* = 0.25, and the high-gain compensator has a gain $G_{P}=1.19$ with a pole located at ω*_Z_* = 17,857 rad/s. The pole of the low-pass filter is located at ω*_P_* = 314,259 rad/s. In the outer voltage loop: the sensor gain value is *H* = 0.15, and the PI-controller has a gain of $K_{P}\;$ = 0.1 with an integrative time of $T_{i}\;$= 350 µs. The high inrush current produced when the converter is initially powered up, can be avoided using an RC circuit to feed the reference voltage $v_{REF}$.

### 6.1. Open Loop Test

**Figure 11:**
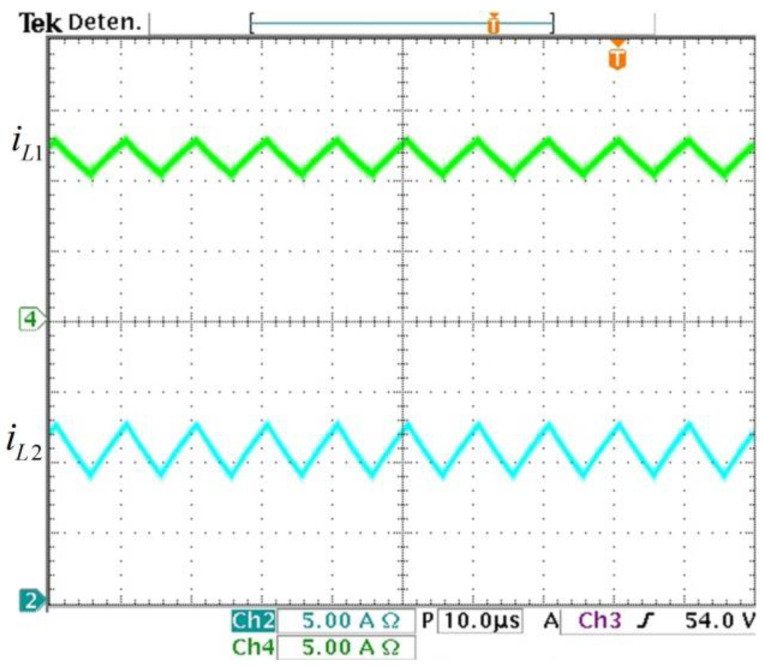
Experimental inductor currents. (From top to bottom) Inductor current *i_L1_* (*y*-axis: 5 A/div) and inductor current *i_L2_* (*y*-axis: 5 A/div) (*x*-axis: time 10 µs/div).

The converter prototype is tested in an open loop to validate the steady-state expressions, transfer functions, and the behavior to load changes. The steady-state values of the prototype are computed according to their corresponding expression: $I_{L1}=10.41$ A, $I_{L2}=10.41$ A, $V_{C1}=48$ V, and *V_O_* = 48 V. Now, measuring the steady-state values from the prototype in the laboratory, the experimental inductor currents are shown in Figure 11. The average inductor currents are: $I_{L1}=11.5\;$ A and $I_{L2}=10.41\;$ A with 20% and 30% of ripples, respectively. The experimental value $I_{L1}$ slightly differs from its theoretical value because of the parasitic elements.

**Figure 12:**
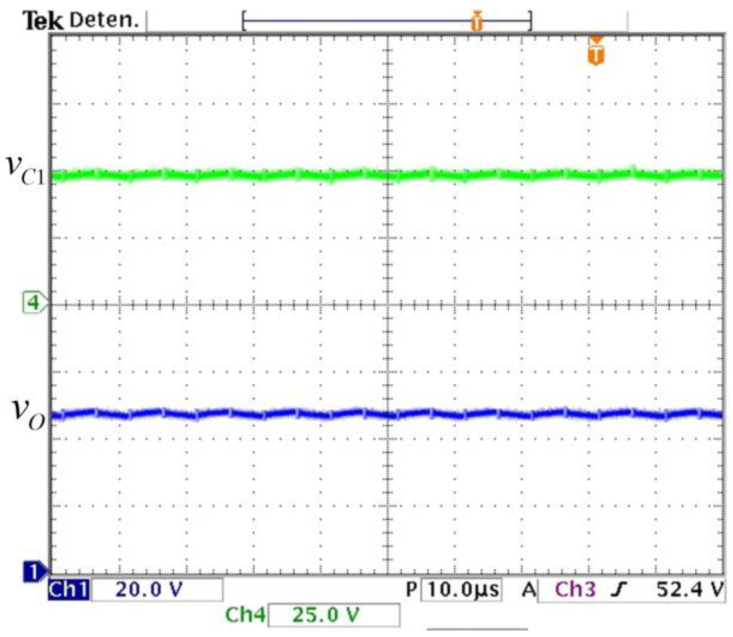
Experimental capacitor voltages. (From top to bottom) Capacitor voltage $v_{C1}$ (*y*-axis: 25 V/div) and output voltage $v_{O}$ (*y*-axis: 20 V/div) (*x*-axis: time 10 µs/div).

**Figure 13:**
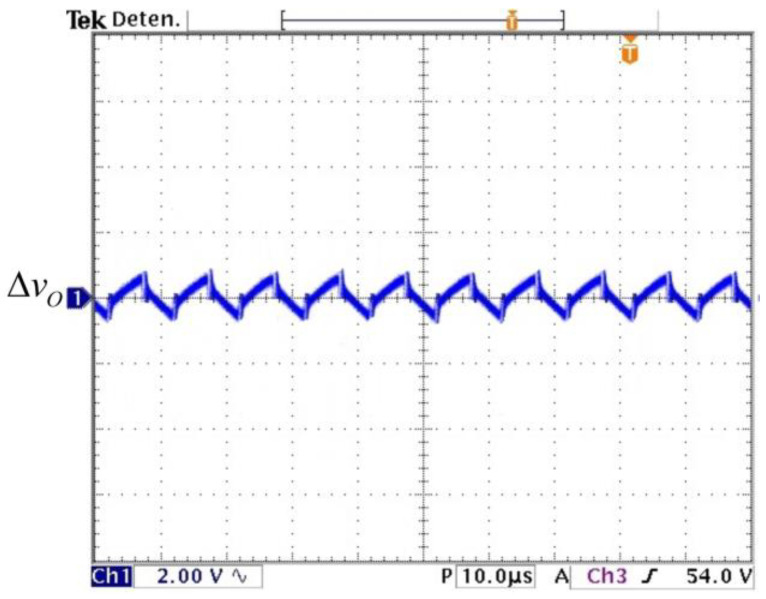
Experimental voltage ripple of output voltage (*y*-axis: 2 V/div) (*x*-axis: time 10 µs/div).

**Figure 14:**
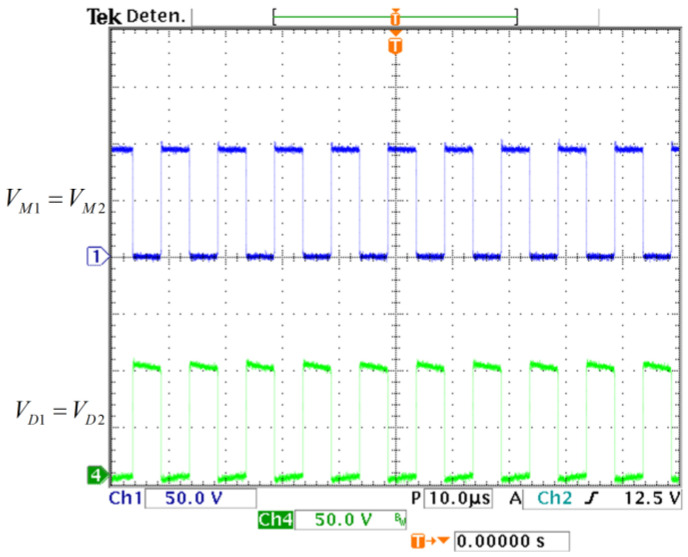
Experimental voltage on semiconductors. (From top to bottom) Stress voltage in MOSFETs (*y*-axis: 50 V/div) and stress voltage in diodes (*y*-axis: 50 V/div) (*x*-axis: time 10 µs/div).

The experimental capacitor voltages are shown in Figure 12; they match the calculated values. A zoom for the voltage $v_{O}$ is measured to watch its ripple (see Figure 13) and it matches with the required 2% peak-to-peak ripple. These results validate the calculated design relationships. The experimental voltage values on the semiconductor switching devices are shown in Figure 14; they verify the expected stress calculated by the expressions obtained in Section 2.

**Figure 15:**
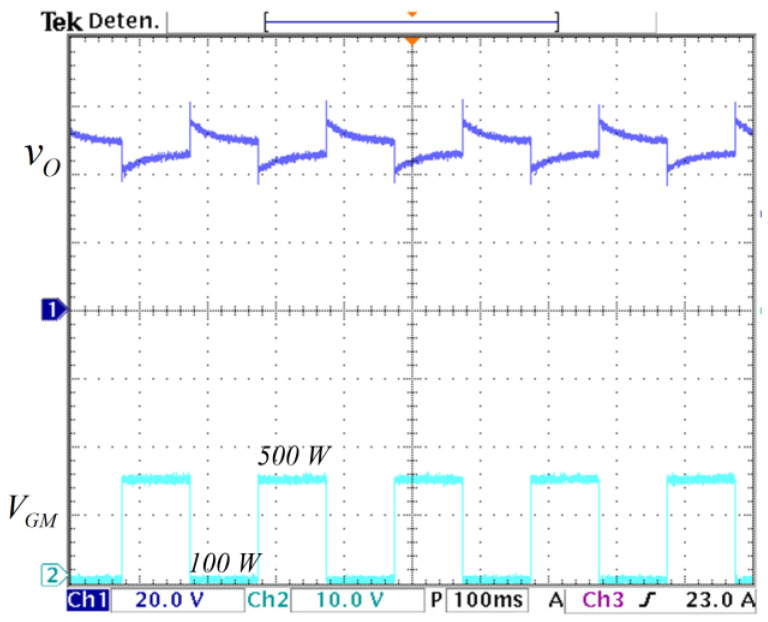
Open-loop response to step changes in the load: (**top**) output voltage of converter $v_{C2}=v_{O}$ (*y*-axis: 20 V/div), and (**bottom**) $V_{GM}$ gate voltage of the MOSFET $M_{C}$ that changes the load from 100 W to 500 W (*y*-axis: 10 V/div) (*x*-axis: time 100 ms/div).

The converter is tested in an open loop to step changes in the load. The circuit used is depicted in Figure 10 inside the block called load. The load changes from $R_{1}||R_{2}$ = 4.6 Ω (500 W) when the MOSFET $M_{C}$ is ON to $R_{1}$ = 23 Ω (100 W) when the MOSFET $M_{C}$ is OFF at a frequency of 5 Hz. The output voltage response to those changes is shown in Figure 15. It can be noticed that the output voltage varies when load changes occur. Therefore, a controller is needed to attenuate those variations.

**Figure 16:**
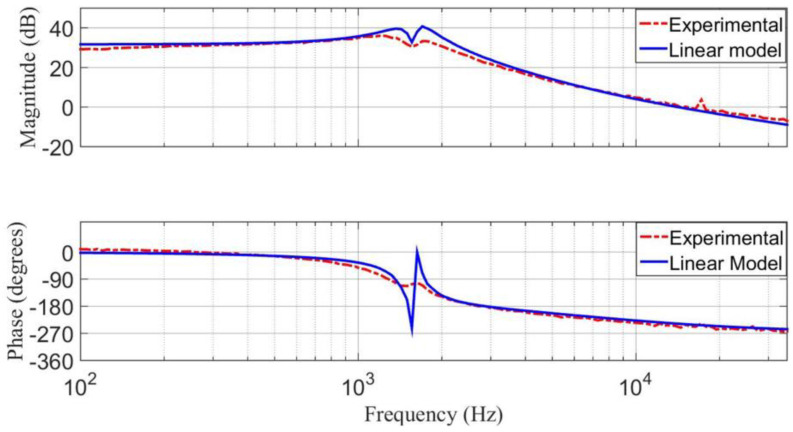
Bode diagram of the transfer function ${\tilde{v}}_{O}(s)/\tilde{d}(s)$ using the linear model and from the experimental frequency response: (**top**) magnitude (*y*-axis: 20 dB/div), and (**bottom**) phase margin (*y*-axis: 90 deg/div).

Using the Frequency Response Analyzer 300 from AP Instruments, an experimental response of the transfer function ${\tilde{v}}_{O}(s)/\tilde{d}(s)$ is obtained for the prototype. It is compared with the response obtained from the linear model using MATLAB software, as shown in Figure 16. The plot of the experimental response (dotted line) is smoother due to the effect of parasitic elements of the step-down/up converter. The parasitic elements were neglected in the linear model (continuous line). The similar behavior of these responses validates the approximation of the linear model of the step-down/up converter.

### 6.2. Closed-Loop Test

**Figure 17:**
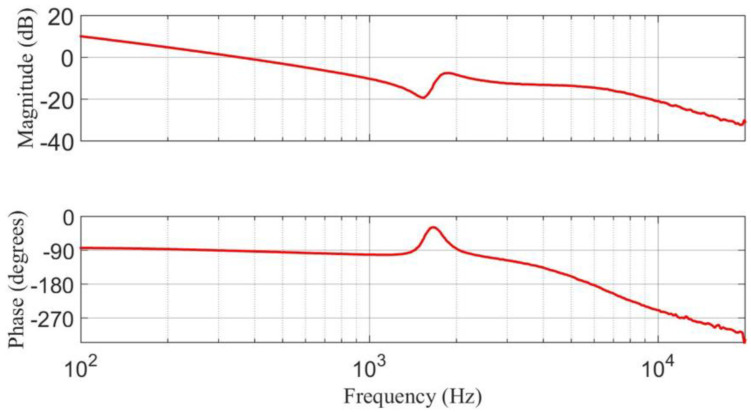
Experimental voltage loop gain at nominal load: (**top**) magnitude (*y*-axis: 20 dB/div), and (**bottom**) phase margin (*y*-axis: 90 deg/div).

When the voltage loop is closed, it is crucial to guarantee an adequate gain margin, phase margin, and crossover slope at 0 dB of the voltage loop gain. Using the Frequency Response Analyzer 300, the experimental frequency response of the voltage loop gain is obtained and depicted in Figure 17. It exhibits a crossover slope at 0 dB of about 20 dB/dec, a phase margin of 90 degrees, and a gain margin of 8 dB. Thus, robust stability is achieved in the switching regulator.

**Figure 18:**
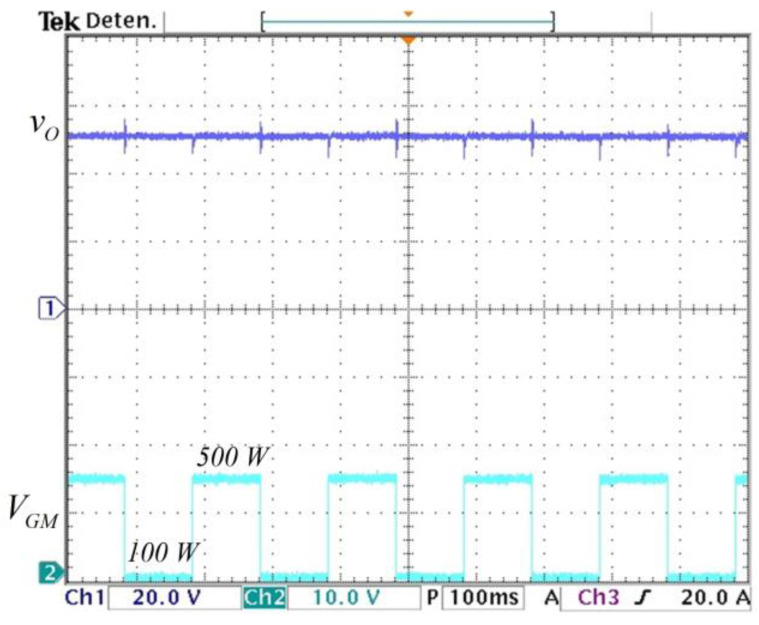
Closed-loop response to step changes in the load: (**top**) output voltage of converter $v_{O}$ (*y*-axis: 20 V/div), and (**bottom**) $V_{GM}$ gate voltage of the MOSFET $M_{C}$ that changes the load from 100 W to 500 W (*y*-axis: 10 V/div) (*x*-axis: time 100 ms/div).

**Figure 19:**
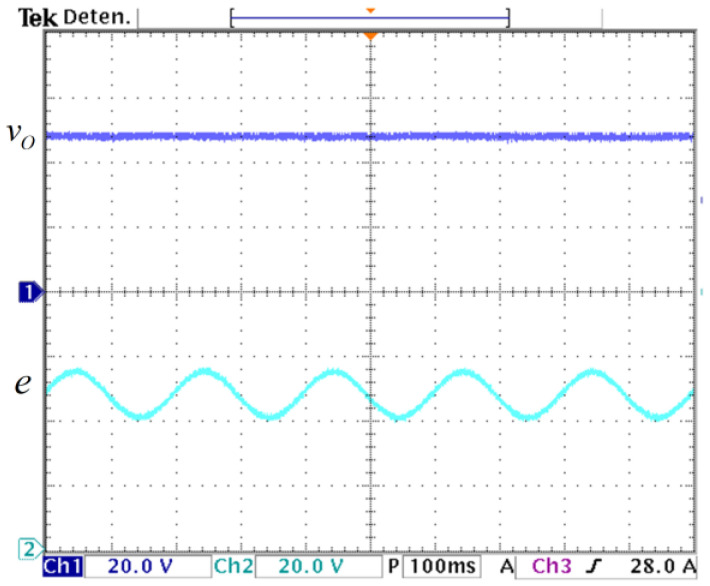
Output voltage response to input voltage variation: (**top**) output voltage $v_{O}$ (*y*-axis: 20 V/div), and (**bottom**) input voltage variation *e* (*y*-axis: 20 V/div), (*x*-axis: time 100 ms/div).

With the controller enabled, the load was again changed from R_1_||R_2_ = 4.6 Ω (500 W) when the M_C_ MOSFET is ON to R_1_ = 23 Ω (100 W) when the M_C_ MOSFET is OFF at a frequency of 5 Hz. The controller maintains the output voltage regulated despite load variations, as it is shown in Figure 18. Furthermore, a fluctuation in the input voltage from 40 V to 56 V is applied to show the behavior of the controller with input voltage fluctuations, as shown in Figure 19. It can be noticed that the controller maintains the output voltage regulated at 48 V despite changes in the input voltage.

**Figure 20:**
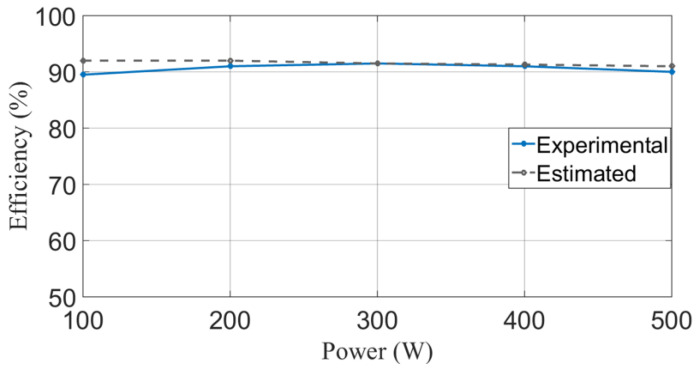
The efficiency of the step-down/up converter for different loads.

Finally, the estimated and experimental efficiencies for this converter were computed. The parasitic elements used to calculate efficiency are shown in the second column of Table 7. These values were obtained from the manufacturer’s datasheet of each component, whose serial number is shown in Table 4. Then, the individual losses are computed using the expressions obtained in Section 5. Finally, the results are shown in the third column of Table 6. Moreover, the power core losses are included (P_LMC_). According to the sum of the individual losses, the total power loss for the prototype is 49.57 W for a power output of 500 W. Evaluating the expression (23), the corresponding estimated efficiency is $\eta_{cal}$ = 91%. Using a procedure similar to the one developed in Table 7, calculations were made to obtain the power losses and the estimated power efficiency from 100 W to 500 W; the results are shown in Table 8. Then, the experimental efficiency is obtained by measuring the input and the output power from the prototype at different loads, and it is compared with the estimated efficiency, as shown in Figure 20. It can be noticed that, in a full load (500 W), the experimental efficiency is 90.5%. Therefore, the estimated efficiency gives a good approximation of the experimental efficiency. Most of the power losses are in the active switches. Moreover, as can be seen, the efficiency remains almost the same for the suggested power range.

## 7. Concluding Remarks

This paper discusses a switching regulator based on a non-inverter output voltage converter, consisting of the non-cascading connection of two converters where part of the energy is processed by only one converter. The converter has two active switches that operate simultaneously with the same duty cycle. The proposed topology can be used when stepping up and down characteristics are required. This converter is suitable when the regulator works as an interface between a lithium-ion battery pack and a load. An improvement in the lifetime of the LIBs will result due to the non-pulsating input current of the converter. The expressions to find the values of circuit elements for this converter are given. For dynamic analysis, the transfer functions of the linear models were derived. A procedure based on loop-shaping techniques was used to design a 500 W regulator for an output voltage of 48 V. The prototype was tested in an open loop and a closed loop. Plots of the steady-state values, output voltage ripple, frequency responses, and input disturbance rejection were obtained. The experimental results validate the steady-state expressions; furthermore, they demonstrate the proper operation of the converter. Responses to load changes and input voltage variations validate the design of the controller. The estimation of the efficiency of the step-down/up converter approximates the experimental efficiency quite well.

## Figures and Tables

**Table 1:** Expressions to design the components of the converter.

Component	Relationship
$L_{1}$	$\frac{{\left(1-D\right)}^{2}R}{2\varepsilon Df_{S}}$
$L_{2}$	$\frac{{\left(1-D\right)}^{2}R}{2\varepsilon f_{S}}$
$C_{1}$	$\frac{D^{2}R}{2\varepsilon (1-D)Rf_{S}}$
$C_{2}$	$\frac{D}{2\varepsilon Rf_{S}}$

**Table 2:** Individual power loss equations.

Component	Power Loss Equation
$L_{1}$	$P_{L\_L1}=I_{L1}^{2}R_{L1}$
$L_{2}$	$P_{L\_L2}=I_{L2}^{2}R_{L2}$
$C_{1}$	$P_{L\_C1}=I_{C1RMS}^{2}R_{C1}$
$C_{2}$	$P_{L\_C2}=I_{C2RMS}^{2}R_{C2}$
$D_{1}$	$P_{L\_D1}=V_{FD1}I_{D1}$
$D_{2}$	$P_{L\_D2}=V_{FD2}I_{D2}$
$M_{1}$	$P_{L\_M1}=\frac{I_{M1}^{2}}{D}R_{M1}+\frac{1}{2}V_{M1}\frac{I_{M1}}{D}\left(t_{rr1}+t_{ff1}\right)f_{S}$
$M_{2}$	$P_{L\_M2}=\frac{I_{M2}^{2}}{D}R_{M2}+\frac{1}{2}V_{M2}\frac{I_{M2}}{D}\left(t_{rr2}+t_{ff2}\right)f_{S}$

**Table 3:** Comparison between proposed converter and other configurations.

Reference	Voltage Gain	Number of Components	Efficiency	Continuous Input Current
[9]	*D*/(1 − *D*)	Switches 4 Diodes 2 Inductors 1 Capacitors 1	*η* = 85.5 *E* = 2.5–4.5 V *V_O_* = 3.3 V *P* = 1 W	No
[10]	*D*/(1.5 − *D*)	Switches 6 Diodes 0 Inductors 3 Capacitors 1	*η* = 95.7 *E* = 2.7–4.2 V *V_O_* = 3.3 V *P* = 1 W	Yes
[12]	*D*/2(1 − *D*)	Switches 1 Diodes 2 Inductors 2 Capacitors 3	*η* = 90% *E* = 17.5–24 V *V_O_* = 21 V *P* = 120 W	Yes
[23]	*D*/(1 − *D*)	Switches 4 Diodes 1 Inductors 1 Capacitors 1	*η* = 95.7 *E* = 48 V *V_O_* = 5–75 V *P* = 35 W	Yes
[24]	*D*/(1 − *D*)	Switches 4 Diodes 1 Inductors 5 Capacitors 5	*η* = 88/94 *E* = 90 V *V_O_* = 90 V *P* = 100/350 W	No
[25]	*D*/(1 − *D*)	Switches 4 Diodes 0 Inductors 3 Capacitors 3	*η* = 88.7/90.4 *E* = 44–60 V *V_O_* = 48 V *P* = 60 W	No
[30]	*D*/(1 − *D*)^2^	Switches 2 Diodes 2 Inductors 3 Capacitors 3	*η* = 92% *E* = 25 V *V_O_* = 100 V *P* = 100 W	Yes
[31]	*D*/(1 − *D*)^2^	Switches 2 Diodes 2 Inductors 2 Capacitors 2	*η* = 91.4% *E* = 36 V *V_O_* = 215 V *P* = 250 W	Yes
Proposed	*D*/(1 − *D*)	Switches 2 Diodes 2 Inductors 2 Capacitors 2	*η* = 90.5% *E* = 40–56 V *V_O_* = 48 V *P* = 500 W	Yes

**Table 4:** Parameters of the converter.

Parameter	Value
*E*	40–56 V
*V_O_*	48 V
*f_S_*	100 kHz
P	500 W
*R*	4.6 Ω
D	0.5
$\Delta I_{L1}$	$0.2I_{L1}$ (20% peak to peak ripple)
$\Delta I_{L2}$	$0.3I_{L2}$ (30% peak to peak ripple)
$\Delta V_{C1}$	$0.02V_{C1}$ (2% peak to peak ripple)
$\Delta V_{C2}$	$0.02V_{C2}$ (2% peak to peak ripple)

**Table 5:** Component values.

Component	Value	Serial Number
*L*_1_	120 µH	1140121KRC
*L*_2_	82 µH	1140820KRC
*C*_1_	56 µF	RNU1J560MDN1PH
*C*_2_	56 µF	RNU1J560MDN1PH
*D*_1_	$I_{D1}=5.2A,\;V_{D1}=96\;V$	DSSK30018A
*D*_2_	$I_{D2}=5.2\;A,\;V_{D2}=96\;V$	DSSK30018A
*M_1_*	$I_{M1}=5.2\;A,\;V_{M1}=96\;V\;$	IRFP4668
*M_2_*	$I_{M2}=5.2\;A,\;V_{M2}=96\;V$	IRFP4668

**Table 6:** Location of poles and zeros of the transfer functions.

Transfer Function	Poles	Zeros
$\frac{{\tilde{i}}_{L1}(s)}{\tilde{d}(s)}$	$\left\{\begin{array}{l} -686\pm 10188i\\ -1327\pm 9544i \end{array}\right\}$	$\left\{\begin{array}{l} -7704\;\\ -174\pm 11201i \end{array}\right\}$
$\frac{{\tilde{v}}_{O}(s)}{\tilde{d}(s)}$	$\left\{\begin{array}{l} -686\pm 10188i\\ -1327\pm 9544i \end{array}\right\}$	$\left\{\begin{array}{l} 49407\;\\ 232\pm 9864i \end{array}\right\}$

**Table 7:** The power losses of parasitic elements at 500 W.

Parasitic Element	Value	Power Loss
$R_{L1}$	28 mΩ	$P_{L\_L1}$ = 3 W
		$P_{LMC1}=\;0.060$ W
$R_{L2}$	23 mΩ	$P_{L\_L2}$ = 2.5 W
		$P_{LMC1}=\;0.050$ W
$R_{C1}$	25 mΩ	$P_{L\_C1}$ = 2.7 W
$R_{C2}$	25 mΩ	$P_{L\_C2}$ = 2.7 W
$V_{FD1}$	0.88 V	$P_{L\_D1}$ = 4.58 W
$V_{FD2}$	0.88 V	$P_{L\_D2}$ = 4.58 W
$R_{M1}$	9.7 mΩ	$P_{L\_M1}$ = 14.7 W
$t_{rr1}$	146 ns	
$t_{ff1}$	138 ns	
$R_{M2}$	9.7 mΩ	$P_{L\_M1}$ = 14.7 W
$t_{rr2}$	146 ns	
$t_{ff2}$	138 ns	
Total Power loss $P_{L\_T}=49.57\;W$		

**Table 8:** Estimated power losses and efficiency.

Power	100 W	200 W	300 W	400 W	500
Power loss	8.55 W	16.95 W	26.9	37.81	49.56
Efficiency (%)	92.1	92.2	91.7	91.3	90.9

## Data Availability

The data that support the findings of this study are available from the corresponding author, upon reasonable request.

## References

[1] Beltrán C.A., Diaz-Saldierna L.H., Langarica-Cordoba D., Martinez-Rodriguez P.R. (2023). Passivity-Based Control for Output Voltage Regulation in a Fuel Cell/Boost Converter System. Micromachines.

[2] Qin Y., Li S., Hui S.Y. (2017). Topology-Transition Control For Wide-Input-Voltage-Range Efficiency Improvement and Fast Current Regulation in Automotive LED Applications. IEEE Trans. Ind. Electron..

[3] Cavallo A., Russo A., Canciello G. (2019). Hierarchical control for generator and battery in the more electric aircraft. Sci. China Inf. Sci..

[4] Canciello G., Cavallo A., Lo Schiavo A., Russo A. (2020). Multi-objective adaptive sliding manifold control for More Electric Aircraft. ISA Trans..

[5] Kim K.D., Lee H.M., Hong S.W., Cho G.H. (2019). A noninverting buck–boost converter with state-based current control for Li-ion battery management in mobile applications. IEEE Trans. Ind. Electron..

[6] Geng Z., Hong T., Qi K., Ambrosio J., Gu D. (2018). Modular regenerative emulation system for DC–DC converters in hybrid fuel cell vehicle applications. IEEE Trans. Veh. Technol..

[7] Ren X., Tang Z., Ruan X., Wei J., Hua G. Four Switch Buck-Boost Converter for Telecom DC-DC Power Supply Applications. Proceedings of the 2008 Twenty-Third Annual IEEE Applied Power Electronics Conference and Exposition.

[8] Tsai C., Tsai Y., Liu H. (2015). A stable mode-transition technique for a digitally controlled non-inverting buck–boost DC–DC converter. IEEE Trans. Ind. Electron..

[9] Tsai Y.-Y., Tsai Y.-S., Tsai C.-W., Tsai C.-H. (2017). Digital noninverting-buck–boost converter with enhanced duty-cycle-overlap control. IEEE Trans. Circuits Syst. II Express Briefs.

[10] Mishra A., De Smedt V. A Novel Hybrid Buck-Boost Converter Topology for Li-ion Batteries with Increased Efficiency. Proceedings of the 27th IEEE International Conference on Electronics, Circuits and Systems (ICECS).

[11] Wei C.-L., Wu C.-H., Wu L.-Y., Shih M.-H. (2010). An Integrated Step-Up/Step-Down DC–DC Converter Implemented With Switched-Capacitor Circuits. IEEE Trans. Circuits Syst. II Express Briefs.

[12] Villanueva-Loredo J.A., Ortiz-Lopez M.G., Leyva-Ramos J., Diaz-Saldierna L.H. (2021). Switching regulator based on switched-inductor SEPIC DC-DC converter with a continuous input current for lithium-ion batteries. IET Power Electron..

[13] Houache M.S.E., Yim C.-H., Karkar Z., Abu-Lebdeh Y. (2022). On the Current and Future Outlook of Battery Chemistries for Electric Vehicles—Mini Review. Batteries.

[14] Wu Y. (2015). Lithium-Ion Batteries: Fundamentals and Applications.

[15] Ahmed M.H., Lee F.C., Li Q. (2021). Two-stage 48-V VRM with intermediate bus voltage optimization for data centers. IEEE J. Emerg. Sel. Top. Power Electron..

[16] Hao L., Namuduri C.S., Gopalakrishnan S., Lee C.J., Shidore N.S., Pandi M., Vandermeir T. (2020). Brushless fast starter for automotive engine start/stop application. IEEE Trans. Ind. Appl..

[17] Selvanathan K., Govindarajan U. (2021). A novel tri-capacity battery charger topology for low-voltage DC residential nanogrid. IET Renew. Power Gener..

[18] Duan C., Wang C., Li Z., Chen J., Wang S., Snyder A., Jiang C. (2018). A solar power-assisted battery balancing system for electric vehicles. IEEE Trans. Transp. Electrif..

[19] Wildfeuer L., Gieler P., Karger A. (2021). Combining the Distribution of Relaxation Times from EIS and Time-Domain Data for Parameterizing Equivalent Circuit Models of Lithium-Ion Batteries. Batteries.

[20] Brand M.J., Hofmann M.H., Schuster S.S., Keil P., Jossen A. (2018). The influence of current ripples on the lifetime of lithium-ion batteries. IEEE Trans. Veh. Technol..

[21] Savoye F., Venet P., Millet M., Groot J. (2012). Impact of periodic current pulses on Li-Ion battery performance. IEEE Trans. Ind. Electron..

[22] Krein P. (2014). Elements of Power Electronics.

[23] Wei A., Lehman B., Bowhers W., Amirabadi M. A soft-switching non-inverting buck-boost converter. Proceedings of the 2021 IEEE Applied Power Electronics Conference and Exposition (APEC).

[24] Cheng X.-F., Zhang Y., Yin C. (2019). A Zero Voltage Switching Topology for Non-Inverting Buck–Boost Converter. IEEE Trans. Circuits Syst. II Express Briefs.

[25] Xue J., Lee H. (2015). A 2-MHz 60-W Zero-Voltage-Switching Synchronous Noninverting Buck–Boost Converter With Reduced Component Values. IEEE Trans. Circuits Syst. II Express Briefs.

[26] Wu K.-C., Wu H.-H., Wei C.-L. (2015). Analysis and Design of Mixed-Mode Operation for Noninverting Buck–Boost DC–DC Converter. IEEE Trans. Circuits Syst. II Express Briefs.

[27] Zarkov Z., Lazarov V., Bachev I., Stoyanov L. Theoretical and Experimental Study of Interleaved Non-Inverting Buck-Boost Converter for RES. Proceedings of the 2018 International Conference on High Technology for Sustainable Development (HiTech).

[28] Alajmi B.N., Marei M.I., Abdelsalam I., Ahmed N.A. (2022). Multiphase Interleaved Converter Based on Cascaded Non-Inverting Buck-Boost Converter. IEEE Access.

[29] Chen J., Maksimović D., Erickson R.W. (2006). Analysis and design of a low-stress buck-boost converter in universal-input PFC applications. IEEE Trans. Power Electron..

[30] Gholizadeh H., Gorji S.A., Sera D.A. (2023). Quadratic Buck-Boost Converter With Continuous Input and Output Currents. IEEE Access.

[31] Mayo-Maldonado J.C., Valdez-Resendiz J.E., Garcia-Vite P.M., Rosas-Caro J.C., del Rosario Rivera-Espinosa M., Valderrabano-Gonzalez A. (2019). Quadratic Buck–Boost Converter with Zero Output Voltage Ripple at a Selectable Operating Point. IEEE Trans. Ind. Appl..

[32] Tse C.K., Chow M.H.L. (2000). Theoretical study of switching power converters with power factor correction and output regulation. IEEE Trans. Circuits Syst. I.

[33] Loera-Palomo R., Morales-Saldaña J.A., Palacios-Hernández E. (2013). Quadratic step-down dc–dc converters based on reduced redundant power processing approach. IET Power Electron..

[34] Zogogianni C.G., Tatakis E.C., Vekic M.S. (2019). Non-Isolated Reduced Redundant Power Processing DC/DC Converters: A Systematic Study of Topologies With Wide Voltage Ratio for High-Power Applications. IEEE Trans. Power Electron..

[35] Leyva-Ramos J., Villanueva-Loredo J.A., Ortiz-Lopez M.G., Diaz-Saldierna L.H. A Non-Cascading Step-up/down DC-DC Converter with Non-Pulsating input Current for Lithium-ion Battery Applications: Analysis and Design. Proceedings of the 2022 IEEE Applied Power Electronics Conference and Exposition (APEC).

[36] Kassakian J.G., Schlecht M.F., Verghese G.C. (2010). Principles of Power Electronics.

[37] Astrom K.J., Murray R.M. (2008). Feedback Systems: An Introduction for Scientist and Engineers.

[38] López-Santos O., Martínez-Salamero L., García G., Valderrama-Blavi H., Mercuri D.O. (2013). Efficiency analysis of a sliding-mode controlled quadratic boost converter. IET Power Electron..

[39] Ridley R., Nace A. Modeling Ferrite Core Losses. https://www.ridleyengineering.com.

[40] Górecki K., Detka K. Analysis of influence of losses in the core of the inductor on parameters of the buck converter. Proceedings of the 2018 Baltic URSI Symposium (URSI).

[41] Iqbal J., Ullah M., Khan S.G., Khelifa B., Ćuković S. (2017). Nonlinear control systems—A brief overview of historical and recent advances. Nonlinear Eng..

[42] Leyva-Ramos J., Ortiz-Lopez M.G., Diaz-Saldierna L.H., Martinez-Cruz M. (2011). Average current controlled switching regulators with cascade boost converters. IET Power Electron..

